# Lattice Boltzmann Method for Evaluating Hydraulic Conductivity of Finite Array of Spheres

**DOI:** 10.1100/2012/527618

**Published:** 2012-05-01

**Authors:** Mário A. Camargo, Paulo C. Facin, Luiz F. Pires

**Affiliations:** Laboratory of Soil Physics and Environmental Sciences, Department of Physics, State University of Ponta Grossa (UEPG), 84.030-900 Ponta Grossa, PR, Brazil

## Abstract

The hydraulic conductivity (*K*) represents an important hydrophysical parameter in a porous media. *K* direct measurements, usually demand a lot of work, are expensive and time consuming. Factors such as the media spatial variability, sample size, measurement method, and changes in the sample throughout the experiment directly affect *K* evaluations. One alternative to *K* measurement is computer simulation using the Lattice Boltzmann method (LBM), which can help to minimize problems such as changes in the sample structure during experimental measurements. This work presents *K* experimental and theoretical results (simulated) for three regular finite arrangements of spheres. Experimental measurements were carried out aiming at corroborating the LBM potential to predict *K* once the smallest relative deviation between experimental and simulated results was 1.4%.

## 1. Introduction

Hydraulic conductivity (*K*) is an important parameter in processes of fluid flow in porous media. *K* indicates how easily certain fluid is transported through porous media, and depends on the media properties as well as percolating fluid characteristics. Pore size distribution, type of pores, tortuosity, and connectivity are some of the factors related to the porous media. Regarding the percolating fluid, its viscosity (*v*) is the main factor related to *K* measurements [1]. For example, increase in water temperature reduces its viscosity and potentially increases *K*. 

*K* determinations are usually characterized by great variability due to factors such as media spatial variability, sample size, measurement method, changes in the sample throughout the experiment, and others [2]. Therefore, representative determination of *K* requires several measurements and samples. Direct *K* measurements are usually expensive and demand hard and thorough technical work [3].

Theoretical models and numeric simulations which enable *K* measurement from information about the porous media structure might be an interesting alternative to predict this physical parameter [4–7].

One theoretical tool that can be successfully used to predict *K* is the Lattice Boltzmann method (LBM) [8, 9]. The LBM is based on evolution of a relaxation equation for fluid particles distribution function, which is related to density and fluid macroscopic momentum. In the LBM equation, there is input data, which is the relaxation time that is related to the number of time steps so that the thermodynamic equilibrium is reached defining fluid viscosity [10]. The LBM can reproduce the macroscopic behavior of a fluid according to the Navier-Stokes (NS) equation. Relative easiness of computation implementation and numeric stability, in a great variety of flow conditions, makes the LBM ideal for treatment of fluid flow in porous media [11].

The development of LB models had important advances in recent decades; for example, today it is possible to simulate compressible, heat, and multiphase flows [10, 12]. However reliability of these applications has occurred through validations without regard to analytical results, which are usually associated with simplified cases of reality. There are few studies in which the LBM is validated with experimental results and the structure of porous media to be simulated is usually obtained indirectly by techniques (computed tomography or image reconstruction) that have their intrinsic sources of error [9, 11, 13].

LBM has been used for problems in porous media under several aspects [9, 11, 14, 15]. However, such studies have posed extra difficulties to the LBM such as image capture and reconstruction of the media as a representative porous media and results presented take into consideration possible deviations related to these difficulties.

In order to create higher possibility of comparison between results of LBM simulations and experimental results, this work suggests simulating the fluid flow through a layer of spheres. This is due to the fact that spheres have the simplest form to be digitally built and their symmetry enables the control of their superficial irregularity on the results, since by increasing their diameter such irregularities are less perceived by the flow.

We propose an experiment where the construction of the porous media is greatly facilitated—a finite array of spheres. The unique source of error is the roughness (discretized surface) of the sphere, which can be controlled with its diameter increase. So, we present in this work experimental and simulated results of *K* measurements in three porous media constituted of regular arrangements of spheres.

The main objective is to show that the LBM method can be used to evaluate *K* in the arrangements analyzed. This objective was achieved through comparisons between experimental and simulated *K*. The success of results proposed in this work points to the future use of this method in representative measurements of more complex porous media such as soil samples.

## 2. Materials and Methods

### 2.1. Experimental Methods

**Figure 1:**
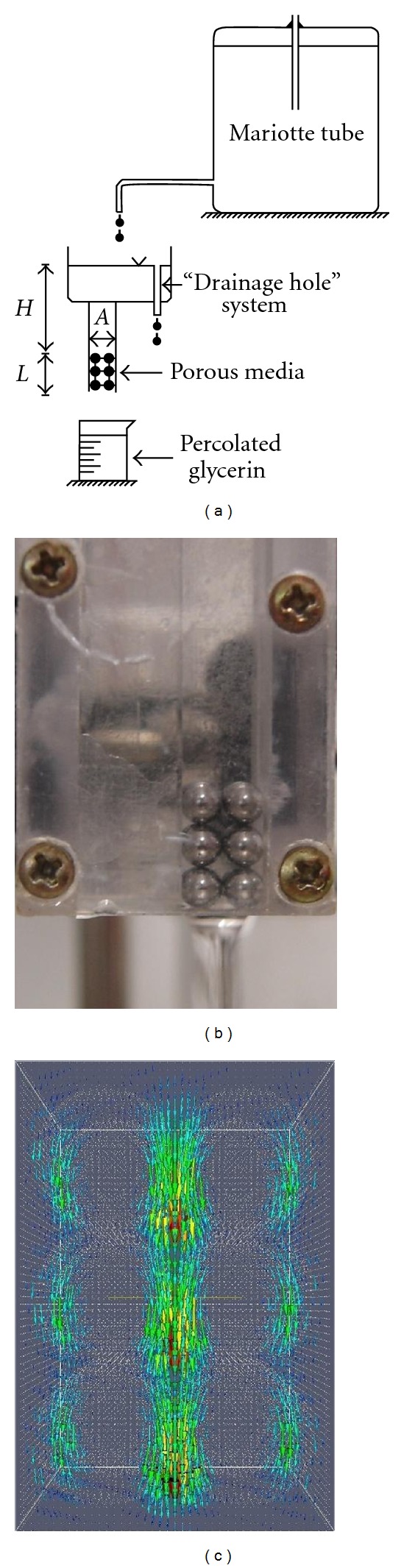
(a) Schematic drawing of the experimental apparatus used; (b) example of a regular arrangement of spheres. The box used to contain the spheres has the following measurements 0.635 cm × 0.635 cm × 4.7 cm (width × height × length) and the diameter of spheres used is 0.3175 cm; (c) velocities field for 3 layers of spheres.

An experimental apparatus tested by Camargo et al. [8] was used for K experimental measurements (Figure 1(a)). The steps below were followed to determine *K*: (a) porous media saturation (acrylic box with a certain sphere arrangement) with glycerin, C_3_H_5_(OH)_3_, (manufactured by Biotec, 99.5% purity); (b) *H* length measurement (hydraulic load) and *L* (porous media height); (c) percolated glycerin mass measurement to obtain its volume, where the glycerin density is known; (d) measurement of the necessary time interval for the glycerin to percolate; (e) use of Darcy's Law to calculate conductivity using

(1) $$K=\left[\frac{V}{\left(At\right)}\right]\left[\frac{L}{\left(L+H\right)}\right],$$

*V* = volume of percolated glycerin (cm^3^), *A* = cross sectional area of the box containing spheres (cm^2^), and *t* = time interval for a given volume of glycerin to percolate (s) [16]; (f) glycerin viscosity measurement using its flow through a *D* = 0.27 cm diameter acrylic cylinder and the analytical expression for the cylinder conductivity *ν* = (*D* ^2^/32)(*g*/*K*_cylinder_): *g* = gravity acceleration (cm s^−2^); (g) *K*_cylinder_ was measured using the steps (a) to (e).

In both *K* measurement cases a sufficiently big *H* was guaranteed so that the glycerin would not form drops when leaving the spheres for the *K* measurement, or the cylinder for the viscosity measurement, which would delay the measurement time used in Darcy's equation.

Glycerin was used because it presents high *ν* leading to a Reynolds number (Re) smaller than 1, where Darcy's Law is valid [17]. Once the glycerin *v* is highly susceptible to temperature variations, its measurement was carried out for each layer of spheres added during the experimental arrangements (Figure 1(b)).

### 2.2. The Lattice Boltzmann Method

**Figure 2:**
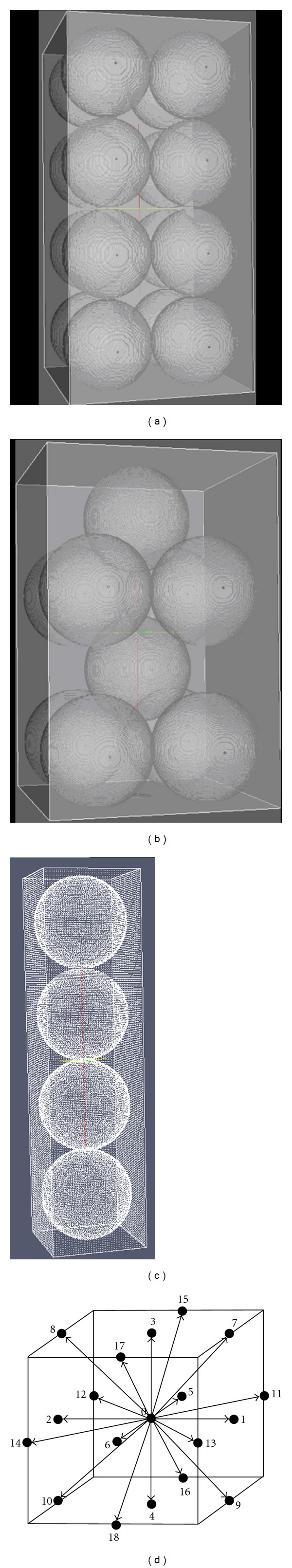
Examples of tridimensional porous media built for the computer simulation (arrangement with four solid layers—SL). (a) QQ; (b) QU; (c) OO and (d) direction vectors of the D3Q19 net site used in simulations [18].

In order to simulate *K* through the LBM a 3D media was built (Figures 2(a)–2(c)) similar to the real porous media being represented in a binary language 0 (solid) and 1 (porous) distributed along the vertices of a regular lattice. Once the porous media was built, the computer program that simulates (2) was used. The program returns the media's intrinsic permeability (*k*) as well as the pressure and flow velocity fields (Figure 1(c)).

The lattice used in the simulations was the cubic D3Q19. The 18 direction vectors of this lattice (Figure 2(d)) connect the sites one to another and also represent the possible velocity vectors, and there is still the null velocity (19 velocities).

Being a site in the lattice located by the vector $\vec{X}$ and having *b*_*m*_ close neighbors, the evolution equation for the particle of fluid distribution function $N_{i}(\vec{X},T)$ is given by the Lattice Boltzmann Equation:

(2) $$N_{i}\left(\vec{X}+{\vec{c}}_{i},T+1\right)=N_{i}\left(\vec{X},T\right)+\Omega_{i}\left(\vec{X},T\right),$$

$\vec{X}$ = vector coordinates of the site in the lattice (lattice units),  *T* = time step variable (0,1, 2,3,…). The duration of a time step is taken to be unity that represents the time interval for the particle of fluid travel between the closest neighbors, $N_{i}(\vec{X},T)$ = number of fluid particles (direction *i*) located at site $\vec{X}$ at time *T*, $\Omega_{i}(\vec{X},T)$ = collision operator that represents the collision of $N_{i}(\vec{X},T)$ fluid particles with others at time *T* (see (4)),  *i* = direction of one of the closest *b*_*m*_ neighbors (0,1, 2,3,…, 19), and ${\vec{c}}_{i}$ = velocity vector in direction *i*. This vector coincides with the lattice vectors, because in a unit time step, a particle travels from one site to adjacent one. The term *i* = 0 represents the *b*_*r*_ resting particles.

Variables $\vec{X}$ and *T* are given in the called lattice units and scale factors are necessaries for these variables assume length (*h*) and time (*δ*) dimensions, that is, it can be assumed that, for example, 5 units of the lattice is equivalent to 1 mm (*h* = 1 mm/5) or 5 time steps equivalent to 1 second (*δ* = 1 s/5). Thus, the scale factor for the velocity vector becomes *h*/*δ*.

In this work we assume that variables without units will be represented as lattice units, that is, length and time variables have as unit the lattice spacing and the time step, respectively. So, velocity, viscosity, pressure, and other properties will be represented by lattice units. 

The mesoscopic dynamics occurs in two steps: (1) propagation step represented by (2); (2) collision step represented by (3), which simulates the molecular collisions needed so that thermodynamic equilibrium occurs. This step is given by the action of collision operator $\Omega_{i}(\vec{X},T)$ on the $N_{i}(\vec{X},T)$:

(3) $$N_{i}^{\prime }\left(\vec{X},T\right)=N_{i}\left(\vec{X},T\right)+\Omega_{i}\left(\vec{X},T\right),$$

$N_{i}^{\prime }(\vec{X},T)$ = “collided” distribution function that will present a new value (number of fluid particles) at site $\vec{X}$, in *i* direction, and time *T*.

A simple and sufficient form of collision operator which recovers the Navier-Stokes macroscopic equation is known as BGK (variables $\vec{X}$ and *T*  were omitted from here to reduce notations) operator [19]:

(4) $$\Omega_{i}=\frac{N_{i}^{\text{eq}}-N_{i}}{\tau },$$

*τ* = relaxation time, which is a function of fluid viscosity, *N*_*i*_^eq^ = equilibrium distribution (see (8)).

So, if *N*_*i*_ < *N*_*i*_^eq^, *Ω*_*i*_ > 0 and the amount *Ω*_*i*_ will be added to *N*_*i*_ making *N*_*i*_ tend to *N*_*i*_^eq^.

The macroscopic particle density and the macroscopic momentum in a site are given by:

(5) $$\overset{b}{\underset{i=0}{\sum }}N_{i}=\rho ,\;\;\overset{b_{m}}{\underset{i=1}{\sum }}N_{i}{\vec{c}}_{i}=\rho \vec{u},$$

*ρ* = density at site $\vec{X}$ at time *T*.

Taking that into consideration, the collision operator conserves mass and momentum,

(6) $$\overset{b}{\underset{i=0}{\sum }}\Omega_{i}=0,\;\;\;\overset{b_{m}}{\underset{i=1}{\sum }}\Omega_{i}{\vec{c}}_{i}=0,$$

where *b* = *b*_*r*_ + *b*_*m*_. 

Particle distribution for the *N*_*i*_^eq^ is usually obtained through the *N*_*i*_^eq^ expansion, in power series at the macroscopic velocity ($\vec{u}$), being *O*(*u* ^2^) sufficient so that Navier-Stokes equation is recovered. For the low Mach number the pressure (*p*) is given by:

(7) $$p=\frac{b_{m}c^{2}}{bD_{e}}\rho ,$$

where *D*_*e*_ is the Euclidian dimension of space in which the lattice is immerse and $c^{2}={\left|{\vec{c}}_{i}\right|}^{2}$. With this, the balance distribution form for moving particles is given by:

(8) $$N_{i}^{\text{eq}}=\frac{\rho }{b}+\frac{\rho D_{e}}{b_{m}c^{2}}c_{i\alpha }u_{\alpha }+\frac{\rho D_{e}\left(D_{e}+2\right)}{2b_{m}c^{4}}c_{i\alpha }u_{\alpha }c_{i\beta }u_{\beta }-\frac{\rho D_{e}}{2b_{m}c^{2}}u^{2}.$$

In the main directions *x*, *y*, and *z*, the balance distribution must be doubled (*N*_*i*_^eq^ = 2*N*_*i*_^eq^) so that viscosity is isotropic [20].

Resting particles have the following balance distribution:

(9) $$N_{o}^{\text{eq}}=\frac{\rho }{b}b_{r}-\frac{\rho }{c^{2}}u^{2}.$$

For a macroscopic analysis of the dynamics proposed by (2), time *δ* and space scales *h* are usually used and the Knudsen variable *k*_*n*_ = *h*/*L* = *δ*/*T*_*c*_ is defined, where *L* and *T*_*c*_ are, respectively, the macroscopic characteristic length and time. With this, the Chapman-Enskog method [20] can be used, considering the equilibrium distribution disturbance, to show that (2) becomes the Navier-Stokes, given by (10), disregarding the O(*k*_*n*_^2^) contributions,

(10) $$\partial_{t}\left(\rho u_{\beta }\right)+\partial_{\alpha }\left(p\right)\delta_{\alpha \beta }+\partial_{\alpha }\left(\rho u_{\alpha }u_{\beta }\right)=v\rho \partial_{\alpha }\left(\partial_{\alpha }u_{\beta }+\partial_{\beta }u_{\alpha }\right),$$

in which *α* and *β* are indexes which represent spatial coordinates *x*, *y,* or *z*; for these indexes Einstein's notation is seen (sum over repeated indexes). Equation (10) is the *β* component of the Navier-Stokes equation with kinematic viscosity *v* = *η*/*ρ* given by:

(11) $$v=\frac{h^{2}c^{2}}{\delta \left(D_{e}+2\right)}\left[\tau -\frac{1}{2}\right].$$

So when the hydrodynamic limit is imposed *L* ≫ *h*, the macroscopic dynamic given by (10) is reproduced by (2).

The boundary conditions used in the simulation are periodic, that is, the fluid which leaves one end of the cavity is injected in the other end. The interaction between fluid and solid occurs so that there is no sliding, in this case, the “bounce back” condition was adopted, where the fluid which collides with the walls has its velocity inverted. The program returns permeability (*k*), which is calculated (12) with the Santos method [11], where in a stationary flow, the strength applied to the fluid is equal to the loss of momentum on the walls:

(12) $$k=v\phi \frac{\langle \text{m}u_{x}\rangle }{{\langle \text{m}u_{x}\rangle }_{\text{lost}}}.$$

*ϕ* = porosity, 〈m*u*_*x*_〉 = fluid average momentum in the porous media, 〈m*u*_*x*_〉_lost_ = fluid average momentum lost in the collisions with the porous media walls in a propagation step.

In order to obtain *K* from *k*, the relation below is used:

(13) $$K=k\frac{g}{v},$$

*k* = permeability, *v* = kinematic viscosity (see (11)).

### 2.3. Computational Simulations

**Figure 3:**
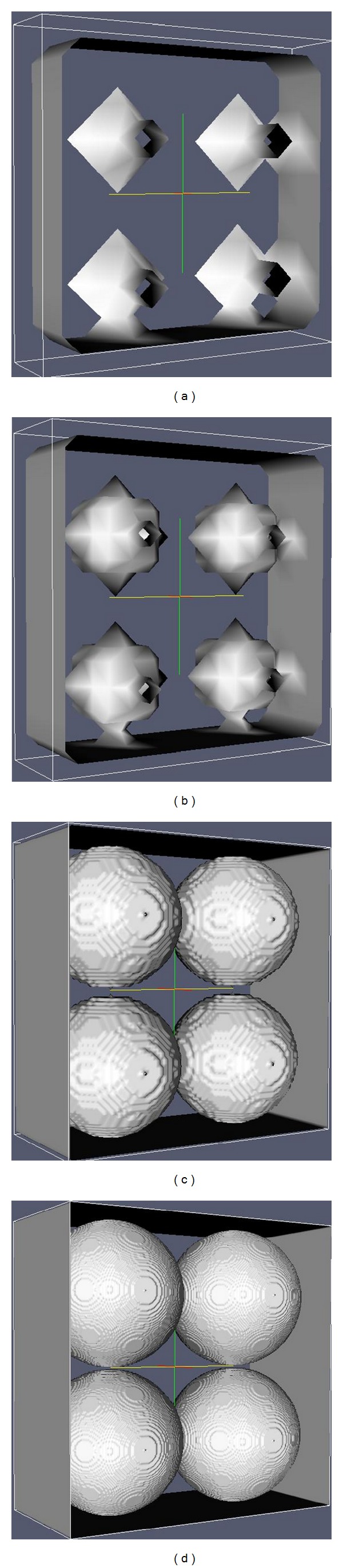
Media built with one solid layer (SL) of four spheres (a) *D* = 1, (b) *D* = 5, (c) *D* = 41, and (d) *D* = 121. The flow occurs coming in (or out) the leaf.

**Figure 4:**
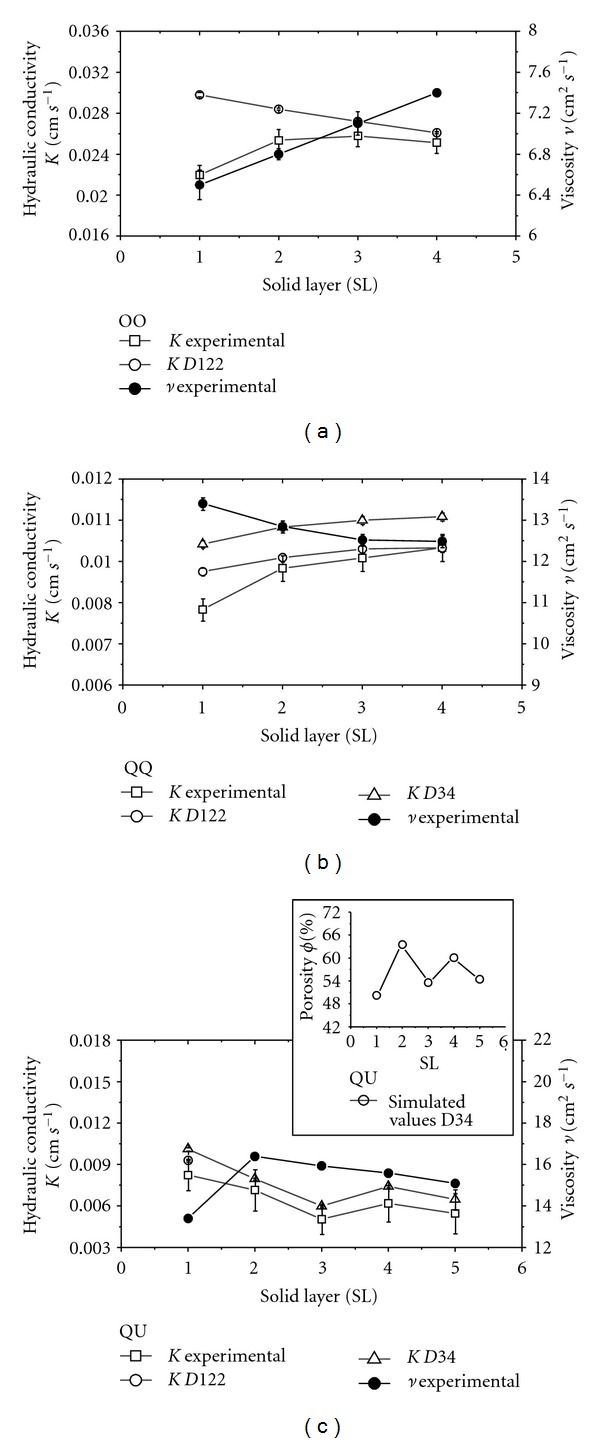
Experimental and simulated results of the hydraulic conductivity (*K*), glycerin experimental viscosity (*v*), and experimental porosity (*ϕ*) for: (a) OO arrangement; (b) QQ arrangement, and (c) QU arrangement. *D*34 and *D*122 represent the spheres diameter (*D*) with 34 and 122 sites.

Different diameters (*D*) of spheres were investigated in the simulations (Figure 3), in order to better compare *K* theoretical results with the ones obtained experimentally (Figure 4).

 The contour conditions used in simulations are periodic and make the fluid that leaves one end of the simulation dominium to enter the other end, as in an infinite array of spheres. Therefore, in the QQ arrangement simulation (Figure 2(a)) the *k* permeability of a single layer of spheres is the same as the permeability of any other number of layers. Despite that, the conductivity might be different, once viscosity is altered by the room temperature in the *K* experimental measurement of each layer.

## 3. Comparison between Experimental and Simulated Results

Larger *D* (Figure 4) provides better approximation between simulated and experimental results. This is due to the reduction in the sphere surface discretization. In the QQ arrangement (Figure 4(b)), porosity is kept constant by the arrangement symmetry, in the QU (Figure 4(c)) the same does not occur and it is necessary to simulate the flow on all sphere layers, which was carried out for *D* = 34 (scale factor *h* = 0.3175 mm/34). Concerning the OO arrangement (Figure 4(a)), the spheres have twice the diameter of the two previous cases. The *K* discrepancy from the first layer to the others remains and is a case to be further investigated.

**Figure 5:**
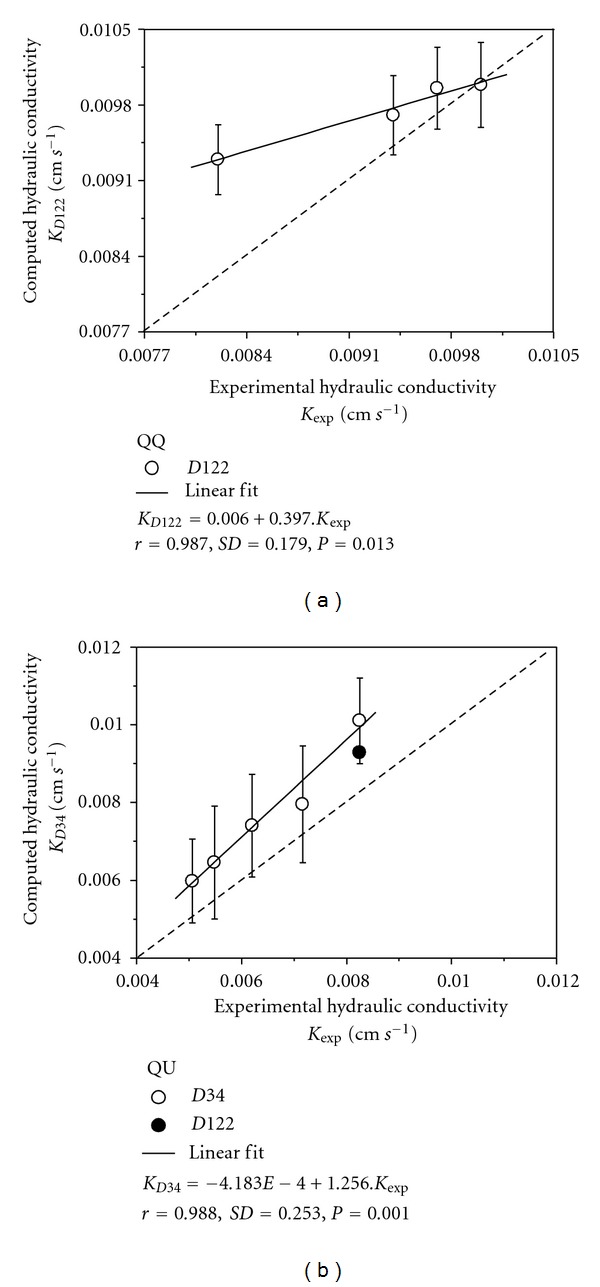
Experimental *K* measured against LBM computer simulated *K* for: (a) QQ arrangement and (b) QU arrangement.

Experimental hydraulic conductivities were plotted against the computed *K* based on the LBM simulations (Figure 5). The data obtained clearly shows that *K* based on computed simulations are in very good agreement (high positive correlation coefficients) with the laboratory measurements. Analyzing the correlation graphics (Figures 5(a) and 5(b)) most of the results are within the variability limits of the laboratory measurements as indicated by the experimental error bars.

In relation to discrepancies found in conductivity values from the first to the other layers, simulations were carried out with a buffer zone up to 100 sites length at the input and output of the flow to check the influence of the periodic contour condition, which does not exist in this experiment. However, no alteration was detected in the conductivities. The interfacial tension might have some effect in the flow output since in the cases in which the hydraulic load is not big enough there is some glycerin dropping. When the hydraulic load is big enough, a stream forms in the flow output, where there are two fluids, glycerin and air; the same does not happen in simulations where there is a monophasic flow. Although there are collision operator models to simulate biphasic flows [10], it is still complicated to control the huge difference between the viscosities of glycerin and air.

Due to some limitation of the PC RAM memory recognition by the software used (~2 Gbytes), it was only possible to simulate one layer of spheres with a maximum diameter *D* = 122 (the same first layer as the QQ case). However, the maximum and minimum deviation for *D* = 34 were 18.6% and 15.5% for QU. It is relevant to point out that the deviations obtained might be minimized with the use of clusters and parallel processing for simulations with larger diameter spheres, mainly in the QU case which represents a more complex media than QQ and OO.

## 4. Concluding Remarks

In this work, an experimental physical reality was pursued with flow in Reynolds low number and spherical symmetry to facilitate the media digital construction, avoiding image acquisition problems. This provided a close comparison between the experimental *K* measurement and the one simulated via LBM, with maximum and minimum deviation (using the experimental value as a reference) from 18.8% and 1.4% for QQ, with the maximum deviation happening only in the first layer. For the OO case, the deviations were 35.6% and 3.9% with the maximum deviation being observed in the first layer again.

In the soil science area, LBM can be used in association with the X-ray computed tomography (CT) utilized to acquire more real 3D soil pore structures. The selection of adequate image analysis procedures, for example, threshold, will allow to accurately reconstructing the pore system structure used to simulate *K* for heterogeneous and nonsymmetrical media such as soil. It is important to mention that no extra computational difficulty is included in this case. 

The use of 3D soil images will make it possible to simulate the 3D fluid flow allowing the evaluation of important hydraulic soil properties such as *k* and *K*. As *K* direct measurements, usually demand a lot of work, are expensive and time consuming, LBM can be an interesting tool to its simulation. With LBM it will also be possible to access the computed flow velocity, which can be utilized for instance to better understand some important phenomena that occur in the soil such as water fingering.
